# Pathogens in Pediatric Septic Arthritis: A Multi-Center Study in Turkiye (PEDSART Study)

**DOI:** 10.3390/children11010134

**Published:** 2024-01-22

**Authors:** Merve Iseri Nepesov, Omer Kilic, Enes Sali, Edanur Yesil, Asuman Akar, Ayse Kaman, Ozge Metin Akcan, Merve Kilic Cil, Canan Ozlu, Sibel Lacinel Gurlevik, Emel Ulusoy, Benhur Sirvan Cetin, Narin Akici, Deniz Cakir, Fatma Deniz Uslu Aygun, Cafer Ozgur Hancerli, Ayse Tekin Yilmaz, Gulsum Alkan, Hatice Uygun, Ibrahim Hakan Bucak, Burcu Bursal, Taylan Celik, Murat Sutcu, Fatma Nur Oz, Zeynep Gokce Gayretli Aydin, Adem Karbuz, Hacer Akturk, Eda Kepenekli, Melike Emiroglu, Selim Oncel, Cagatay Nuhoglu, Ismail Hakki Korucu, Mustafa Incesu, Ahmet Kaya, Hasan Bombaci, Meltem Dinleyici, Kursat Bora Carman, Murat Duman, Ozden Turel, Dilek Yilmaz, Derya Alabaz, Nursen Belet, Gonul Tanir, Mehmet Turgut, Solmaz Celebi, Necdet Kuyucu, Emin Sami Arisoy, Gul Durmaz, Mucahit Kaya, Ates Kara, Ener Cagri Dinleyici

**Affiliations:** 1 Division of Pediatric Infectious Diseases, Department of Pediatrics, Faculty of Medicine, Eskisehir Osmangazi University, Eskisehir 26040, Türkiye; iserimerve@yahoo.com (M.I.N.); omerkilic7@yahoo.com (O.K.); 2 Department of Pediatric Infectious Diseases, Umraniye Training and Research Hospital, Istanbul 34764, Türkiye; 3 Department of Pediatric Infectious Diseases, Mersin City Hospital, Mersin 33240, Türkiye; 4 Division of Pediatric Infectious Diseases, Department of Pediatrics, Faculty of Medicine, Mersin University, Mersin 33110, Türkiye; 5 Department of Pediatric Infectious Diseases, Dr. Sami Ulus Maternity and Children’s Training and Research Hospital, Ankara 06080, Türkiye; 6 Division of Pediatric Infectious Diseases, Department of Pediatrics, Faculty of Medicine, Necmettin Erbakan University, Konya 42090, Türkiye; 7 Department of Pediatric Infectious Diseases, Adana City Hospital, Adana 01230, Türkiye; 8 Division of Pediatric Infectious Diseases, Department of Pediatrics, Faculty of Medicine, Dokuz Eylul University, Izmir 35210, Türkiye; 9 Department of Pediatrics, Faculty of Medicine, Hacettepe University, Ankara 06230, Türkiye; 10 Division of Pediatric Emergency Medicine, Department of Pediatrics, University of Health Sciences Izmir Dr. Behçet Uz Child Disease and Pediatric Surgery Training and Research Hospital, Izmir 35210, Türkiye; 11 Division of Pediatric Infectious Diseases, Department of Pediatrics, Faculty of Medicine, Erciyes University, Kayseri 38030, Türkiye; 12 Department of Pediatrics, University of Health Sciences, Haydarpaşa Numune Training and Research Hospital, Istanbul 34668, Türkiye; 13 Division of Pediatric Infectious Diseases, Department of Pediatrics, Faculty of Medicine, Cerrahpaşa University, Istanbul 34098, Türkiye; 14 Department of Orthopedics and Traumatology, Kanuni Sultan Suleyman Training and Research Hospital, Istanbul 34303, Türkiye; 15 Division of Pediatric Infectious Diseases, Department of Pediatrics, Faculty of Medicine, Kocaeli University, İzmit 41001, Türkiye; 16 Division of Pediatric Infectious Diseases, Department of Pediatrics, Faculty of Medicine, Selcuk University, Konya 42130, Türkiye; 17 Division of Pediatric Infectious Diseases, Department of Pediatrics, Faculty of Medicine, Adıyaman University, Adıyaman 02040, Türkiye; 18 Department of Pediatrics, Kanuni Sultan Suleyman Training and Research Hospital, Istanbul 34303, Türkiye; 19 Division of Pediatric Infectious Diseases, Department of Pediatrics, Faculty of Medicine, Canakkale Onsekiz Mart University, Canakkale 17020, Türkiye; 20 Division of Pediatric Infectious Diseases, Department of Pediatrics, Faculty of Medicine, Istinye University, Istanbul 34010, Türkiye; 21 Division of Pediatric Infectious Diseases, Department of Pediatrics, Faculty of Medicine, Karadeniz Technical University, Trabzon 61080, Türkiye; 22 Department of Pediatric Infectious Diseases, Prof. Dr. Cemil Tascioglu City Hospital, Istanbul 34384, Türkiye; 23 Division of Pediatric Infectious Diseases, Department of Pediatrics, Faculty of Medicine, Koc University, Istanbul 34010, Türkiye; 24 Division of Pediatric Infectious Diseases, Department of Pediatrics, Faculty of Medicine, Marmara University, Istanbul 34854, Türkiye; 25 Department of Orthopedics and Traumatology, Faculty of Medicine, Necmettin Erbakan University, Konya 42090, Türkiye; 26 Department of Orthopedics and Traumatology, University of Health Sciences, Izmir Tepecik Training and Research Hospital, Izmir 35020, Türkiye; 27 Department of Orthopedics and Traumatology, University of Health Sciences, Haydarpaşa Numune Training and Research Hospital, Istanbul 34668, Türkiye; 28 Department of Pediatrics, Faculty of Medicine, Eskisehir Osmangazi University, Eskisehir 26040, Türkiye; 29 Division of Pediatric Emergency Medicine, Department of Pediatrics, Faculty of Medicine, Dokuz Eylul University, Izmir 35160, Türkiye; 30 Division of Pediatric Infectious Diseases, Department of Pediatrics, Faculty of Medicine, Medeniyet University, Istanbul 34700, Türkiye; 31 Division of Pediatric Infectious Diseases, Department of Pediatrics, University of Health Sciences, Izmir Tepecik Training and Research Hospital, Izmir 35020, Türkiye; 32 Division of Pediatric Infectious Diseases, Department of Pediatrics, Faculty of Medicine, Cukurova University, Adana 01330, Türkiye; 33 Division of Pediatric Infectious Diseases, Department of Pediatrics, Faculty of Medicine, Uludag University, Bursa 16059, Türkiye; 34 Department of Microbiology, Faculty of Medicine, Eskişehir Osmangazi University, Eskisehir 26040, Türkiye; 35 Diagen Biotechnological Systems Healthcare and Automation Company, Ankara 06070, Türkiye

**Keywords:** septic arthritis, children, polymerase chain reaction

## Abstract

Objectives: Septic arthritis (SA) is a serious bacterial infection that must be treated efficiently and timely. The large number of culture-negative cases makes local epidemiological data important. Accordingly, this study aimed to evaluate the etiology, clinical characteristics, and therapeutic approach of SA in children in Turkiye, emphasizing the role of real-time polymerase chain reaction (PCR) techniques in the diagnosis. Methods: In this multi-center, prospective study, children hospitalized due to SA between February 2018 and July 2020 in 23 hospitals in 14 cities in Turkiye were included. Clinical, demographic, laboratory, and radiological findings were assessed, and real-time PCR was performed using synovial fluid samples. Results: Seventy-five children aged between 3 and 204 months diagnosed with acute SA were enrolled. Joint pain was the main complaint at admission, and the most commonly involved joints were the knees in 58 patients (77.4%). The combination of synovial fluid culture and real-time PCR detected causative bacteria in 33 patients (44%). In 14 (18.7%) patients, the etiological agent was demonstrated using only PCR. The most commonly isolated etiologic agent was *Staphylococcus aureus*, which was detected in 22 (29.3%) patients, while *Streptococcus pyogenes* was found in 4 (5.3%) patients and *Kingella kingae* in 3 (4%) patients. *Streptococcus pyogenes* and *Kingella kingae* were detected using only PCR. Most patients (81.3%) received combination therapy with multiple agents, and the most commonly used combination was glycopeptides plus third-generation cephalosporin. Conclusions: *Staphylococcus aureus* is the main pathogen in pediatric SA, and with the use of advanced diagnostic approaches, such as real-time PCR, the chance of diagnosis increases, especially in cases due to *Kingella kingae* and *Streptococcus pyogenes.*

## 1. Introduction

Septic arthritis is a serious bacterial joint infection that can lead to significant morbidity and lifelong sequelae [1,2]. The incidence of acute SA is estimated to be 4 to 20 per 100,000 children [3,4,5]. Early and accurate diagnosis and appropriate treatment are needed to prevent complications [1,6]. The detection of the causative microorganism is required to confirm the diagnosis and choose the antimicrobial regimen [7]. Moreover, considering the differences in clinical courses and complication rates between the etiologic pathogens, the identification of the organism will provide important data for patient follow-up.

*Staphylococcus aureus* is the most cultured organism; due to the high rates of negative cultures, the use of molecular diagnostic tests is essential [8]. Recently, PCR techniques have made it much more likely to find the harmful organism. These techniques have also made it easier to identify *Kingella kingae*, the fastidious microorganism that is difficult to isolate on standard agar plates [8,9,10,11].

Currently, there is a lack of data on the causative organisms of pediatric SA in Turkiye. Hence, this study is the first in Turkiye to utilize molecular diagnostic tests in this area.The present study aimed to evaluate the epidemiology, particularly the bacteriologic etiology, and the clinical features of pediatric SA infection in Turkiye. The findings of this study will make a significant contribution to the literature.

## 2. Materials and Methods

### 2.1. Study Design and Population

We prospectively analyzed the clinical and laboratory findings of pediatric SA patients between February 2018 and July 2020 from 23 hospitals in 14 cities. The local ethics committee of Eskisehir Osmangazi University, Faculty of Medicine approved the study (February 2018—decision number 08). Eskisehir Osmangazi University’s Scientific Research Projects (2019/11052) supported the study. Written informed consent was obtained from the parents of all children.

We included all patients who fulfilled the following criteria: (1) patient between 1 month and 18 years; (2) clinical findings compatible with a joint infection (fever, local pain, erythema, swelling, or decreased range of motion); (3) positive synovial fluid culture or detection of the causative agent by PCR from synovial fluid; (4) elevated acute phase reactants, either C-reactive protein (CRP) or erythrocyte sedimentation rate (ESR); (5) imaging findings (like joint effusion) consistent with SA; (6) favorable response to antibiotic treatment with a one-year follow-up [8,12,13,14,15]. During the follow-up period, the patients diagnosed with conditions other than SA were excluded from the study. We excluded children with infections that occurred after surgery or a fracture, children who were carriers of prosthetic materials, and newborns (<1 month of age) from the study.

We analyzed the demographic data (age and sex); clinical information (underlying disease, risk factors, presenting symptoms and duration, affected joint, and treatment modality and duration); routine laboratory tests including white blood cell count (WBC) and differential, ESR, and CRP; microbiological evaluation including blood cultures, synovial fluid cultures, and real-time PCR results. The results of available imaging modalities used, such as X-ray (widening of the joint space, soft tissue swelling), ultrasonography (joint effusion and synovial swelling), computed tomography (CT) (joint effusion, destruction of the subchondral bone), magnetic resonance imaging (MRI) (periarticular high-intensity signal, cartilage destruction), and scintigraphy (increased activity in the early phase and increased bony uptake on both sides of the joint), were also analyzed.

Researchers described sequelae as any undesirable clinical conditions that were observed in the follow-up period after treatment ended and were related to the joint infections [1].

### 2.2. Microbiological Investigations

All the children had joint fluid aspirations to document the infection. The synovial fluid samples collected after receiving consent from the participants were transported to the central laboratory, where real-time PCR analyses were conducted. Real-time PCR analysis targeted the most common pathogens (*Streptococcus pyogenes*, *Streptococcus agalactiae*, *Staphylococcus aureus*, and *Kingella kingae*) causing SA in children.

### 2.3. DNA Extraction

For the DNA extraction protocol, a QuickGene-Mini80 extraction instrument (Kurabo, Osaka City, Japan) was used. First, 100 µL synovial fluid samples were placed into 2 mL microtubes, and 100 µL of PBS (phosphate buffered saline) was added. Subsequently, 30 µL of EDT (proteinase K) and 250 µL LDT (lysis buffer) solution were added and mixed thoroughly by vortexing for 15 s. Then incubate the mixture at 56 °C for two minutes. After the incubation period, 250 µL, or 99%, of cold ethanol was added to the lysate and mixed thoroughly by vortexing for 15 s. Next, we performed the transfer of all contents of the microtube into QuickGene cartridges (Kurabo, Osaka City, Japan), and by following the manufacturer’s instructions, DNA washing and an elution procedure were conducted. Using a 750 µL WDB (wash buffer) solution, DNA washing was performed three times. After performing the extraction protocol, we obtained genomic DNA, which we eluted with 50 µL CDB (elution buffer).

### 2.4. PCR Amplification

Real-time PCR kits, *S. pyogenes* (Vivantis, Cat no. QM2063), *S. aureus* (Vivantis, Cat. No. QM2055), *S. agalactiae* (Vivantis, Cat. No. QM2058), and *K. kingae* (Qiagen, Cat no. BBID00179AR), were used in the study. The specific targets were the collagen-like surface protein (sc1A) genes for *S. pyogenes*, the aminoacyltransferase FemB (femB) gene for *S. aureus*, the cAMP factor gene (cfb) for *S. agalactiae*, and the 16S rRNA gene for *K. kingae*.

Each tube contained a mixture of 15 µL, consisting 10 µL 2× Master mixes, 1 µL Primer/Probe mix, and 4 µL distilled water, prepared in tubes. Additionally 5 µL of targeted DNA was added to each tube. Distilled water was added to the negative control tubes, while the reference strain was added to the positive control tubes. The PCR assay was performed using the Applied Biosystems™ 7500 Real-Time PCR Systems instrument under the following conditions: a first cycle of denaturation at 95 °C for 2 min, followed by 50 cycles of 95 °C for 10 s, and 60 °C for 60 s. We determined the Ct (cycle threshold), which represents the fluorescent signal exhibited by the microorganism in each cycle [16].

We also analyzed all synovial fluid samples for *Neisseria meningitidis*. The specific targets used for *N. meningitidis* were ctrA, porA, and sodC (real-time PCR kit, catalog number: ECD-3101-25hD).

### 2.5. Statistical Analysis

The Statistical Package for Social Science (SPSS) program (Version 15.0, SPSS Inc., Chicago, IL, USA) was used for statistical analysis. The parameters with normal distribution are presented as mean ± standard deviation (SD), and the parameters that did not meet this test are presented as medians along with the interquartile range (CI, 95%). Quantitative variables were expressed as mean (±standard deviation) or median values, while qualitative variables were expressed as percentages.

## 3. Results

The study enrolled seventy-five children diagnose with acute SA, aged between 3 and 204 months. Among them, 45 (60%) were boys and 30 (40%) were girls. Among children, the mean age (±SD) was 86.3 (±57.4) months. The disease season was autumn in 40% (n = 30) of patients, winter in 24% (n = 18) of patients, summer in 22.7% (n = 17), and spring in 13.3% (n = 10) of patients. There was no history of chronic disease in 81.3% (61) of the patients. While 88% (66) of the patients had no history of regular medication use, two patients had a history of anti-tumor necrosis factor drug usage: one of them with a diagnosis of juvenile rheumatoid arthritis and the other one with Crohn’s disease. One of these two patients received concomitant methotrexate therapy, while the other received steroids.

### 3.1. Clinical Data

Upon admission, 84% of patients reported joint pain, 74.6% reported joint swelling, 62.6% reported decreased range of motion, and 36% reported erythema. The median duration of symptoms before admission to the hospital was 4 (1–30) days. Eighty percent of patients had no history of antibiotic use before admission. The average duration of antibiotic therapy was 5.2 days in the group using antibiotics before admission.

The most commonly involved joints were the knee in 58 (77.4%), hip in 11 (14.7%), ankle in four (5.3%), and elbow in one (1.3%). One patient demonstrated multifocal involvement, which includes both the knee and ankle.

The demographic characteristics of the patients are shown in Table 1.

### 3.2. Laboratory Findings and Radiological Investigation

At the time of diagnosis, the median WBC was 12.290 (4.990–38.190) cells/mm^3^, the median CRP value was 88 (4–421) mg/L, and the median ESR was 54 (3–160) mm/h. We also performed a white blood cell count on synovial fluid samples. The median WBC in the synovial fluid sample was 30.642 (1.000–177.300) cells/mm^3^, while the median neutrophil percentage in the joint fluid was 87.5 cells/mm^3^.

All patients underwent at least one radiological imaging including X-ray, ultrasonography, MRI, CT, or scintigraphy. Ultrasonography is the most commonly used method; 58 (77.3%) of the patients underwent ultrasonography imaging. All had findings suggesting SA. 22 out of 44 (58.7%) X-rays detected radiological findings indicative of SA. During the treatment period MRI was usually used, with 38 patients undergoing MRI. All had positive findings. Scans for Tc99m were positive in two out of two (2.7%) patients, and CT in one (1.3%).

### 3.3. Microbiological Investigation

Causative bacteria were detected from a synovial fluid sample via the standard culture method or real-time PCR in 33 (44%) patients out of 75 children. The real-time PCR method alone demonstrated the etiological agent in 14 (18.7%) patients, while both the culture and real-time PCR methods detected the same microorganisms in 12 (16%) patients. In seven (9.3%) patients, the bacteria were cultured, and the real-time PCR results were negative. The most commonly isolated etiologic agent was *S. aureus*. Table 2 shows the microbiological results of the patients.

In our cohort, there were 20 children in the *Kingella* age group (under 48 months). Among them, organisms were detected in none of them by culture; however, in 3, real-time PCR confirmed the presence of *K. kingae*. Bacteremia due to *S. aureus* was seen in 6 patients. Real-time PCR and culture did not detect any microorganisms in the synovial fluid of these four patients, while in one patient, *S. aureus* was detected using the real-time PCR method and culture detected *S. aureus* in one of them. Pathogen distributions according to age group, season, and joint involvement are shown in Table 3. We did not detect *N. meningitidis* in our study population.

### 3.4. Treatment Modality and Follow-Up

Most patients, or 81.3% (n = 61), received combination therapy with multiple agents, and there were 25 different treatment regimens between clinicians. The most commonly used combinations were glycopeptides plus third-generation cephalosporin, taken from 30.7% (n = 23) of patients. Moreover, 78.7% (n = 59) received an antibiotic active against methicillin-resistant *Staphylococcus aureus* (MRSA) empirically. Cephalosporins were the most commonly administered oral antibiotics (32%, n = 24), followed by amoxicillin-clavulanate (24%, n = 18). The median intravenous treatment duration was 14 (2–46) days, while the median oral treatment duration was 12 (0–150) days. Twelve (16%) patients required surgical intervention in addition to antimicrobial treatment. A combination therapy containing glycopeptide (18 patients) and clindamycin (11 patients) was preferred for the treatment of 42 (56%) patients in whom the causative agent could not be isolated, while cephalosporins or ampicillin sulbactam were used in the remaining 13 patients. In these patients, the median intravenous treatment duration was 15 (2–42) days. During the one-year follow-up, these patients did not get any other diagnosis other than SA and had a good response to antibiotic treatment.

We observed concurrent osteomyelitis in 10 out of 75 (13.3%) children. The diagnosis is confirmed by characteristic findings of osteomyelitis on MRI in these 10 patients, while in one patient scintigraphy imaging was also used. Girls account for the majority of osteomyelitis cases (6/10). Five of the patients had a history of chronic illness (asthma, cerebral palsy, hemophilia A, juvenile rheumatoid arthritis, hydronephrosis). *S. aureus* was the causative bacteria in five of them, while *Salmonella* spp. was the causative bacteria in one; the etiological agent could not be detected in four of them. Four children (5.3%) experienced sequelae, specifically limited mobility.

## 4. Discussion

Although SA is a pediatric emergency, its definitive diagnosis remains challenging since the identification of the organism is not possible in most patients. Identifying the organism is not just important for diagnosis but also for guiding the treatment modality. Our study both provides the etiology of pediatric SA cases in Turkiye and includes the supplemental contribution of the real-time PCR method to diagnosis. In studies, the rate of determination of the etiologic causes in synovial fluid by the standard culture method varies between 14.8 and 40% in pediatric cases [1,8,10,17,18,19]. Most SA cases were culture-negative, which can lead to empiric antibiotic failure and require changes in therapy [17]. By using the PCR method, the rate of detecting causative agents increases to 34–67% [8,10,18,19]. 

Our study detected the causative microorganism using real-time PCR in 14 (18.7%) patients with culture negative results, suggesting the importance of nucleic acid amplification tests as a diagnostic procedure in children with SA. The use of PCR on synovial fluid significantly increases the microbiologic diagnosis of SA and shortens the time to identify pathogens. PCR also enhances the identification of organisms that are difficult to culture, like *K. kingae*, and detects the pathogen even after antibiotic therapy [8,9,10,18].

*Staphylococcus aureus* is the most common microorganism causing SA in children [1,18,20]. In our cohort, *S. aureus* was the most common etiologic agent detected via synovial fluid microbiologic investigation in 22 (29.3%) patients, of whom 5 developed osteomyelitis. Moreover, in four of the patients, *S. aureus* was demonstrated via blood culture, while synovial fluid results were negative. In children under the age of 4, *K. kingae*, which is a fastidious, gram-negative coccobacillus bacterium that colonized the oropharynx after the age of 6 months, has been reported more frequently in the etiology of osteoarticular infections after the implementation of PCR techniques in clinical practice [11,18,20,21,22,23,24]. In the literature, the frequency of *K. kingae* probably varies based on the patients’ age or epidemiological differences between countries. Carter et al. [8] reported *K. kingae* in nearly 10% of pediatric SA cases, mainly detected using the PCR method, while Ilharreborde et al. [18] found *K. kingae* in 35% of patients in mainly culture-negative cases. In Ferroni et al.’s [25] study, while 76% of pediatric SA cases under the age of 4 were attributed to *K. kingae*, no cases have been detected above the age of 4. Almost all the SA cases due to *K. kingae* occur in children under 48 months of age [20,23,25]. In our cohort, only 20 (26.6%) children were between 6 and 48 months old, that can contribute to the detection of *K. kingae* to a lesser extent which was 4%.

In our cohort of five patients, either the ESR or CRP value was below 15 mm/h or 15 mg/L. Only one of them took oral amoxicillin-clavulanic acid for two days before admission. Because they presented with an acute monoarthritic clinic and their symptoms could not be explained with another diagnosis, antibiotic treatment was started, and during the follow-up period, a positive clinical response was obtained. In two of these patients, *S. aureus* was demonstrated, one using the real-time PCR method and the other using both the real-time PCR and culture methods. In one of these patients, there was also osteomyelitis. The median duration of symptoms before admission was 2 (1–4) days. Notably, acute phase values may be normal in the early period.

For all patients with suspected SA, a blood culture investigation should be performed. In a multicenter study that included 232 SA cases, 25% of patients had a positive blood culture on admission [1]. Blood culture also provides additional information in cases where the agent cannot be detected from the synovial fluid sample [18]. Four of our patients had negative synovial fluid results for *S. aureus*, as blood culture demonstrated.

Primary meningococcal arthritis is defined as isolated SA without any other finding of invasive meningococcal disease [26,27]. Although it is rarely reported in the literature, using real-time PCR, which is more sensitive than culture in identifying *N. meningitidis*, it is thought that the frequency is higher than what is predicted and diagnosed [26]. In our study, all samples were evaluated using the real-time PCR method for *N. meningitidis,* but no case was detected.

There was a great heterogeneity in the choice of empiric antibiotic therapy between centers. The most important reason for this is the absence of an identifiable pathogen in cultures. For children older than 3 months, it is recommended to use antistaphylococcal penicillin or first-generation cephalosporin as *S. aureus* is the most demonstrated agent. If the country’s local epidemiological result shows a prevalence of MRSA ≥ 10%, antibiotics against MRSA are recommended [7]. In our cohort, 43% of *Staphylococcus aureus* detected in culture and tested for antimicrobial susceptibility were MRSA. 78.7% (n = 59) of patients were given an antibiotic that was proven to be effective against MRSA. At this point, antibiotic treatment should be based on local microbiological epidemiology. Still, the lack of study on the etiology of pediatric SA in Turkiye, especially after the addition of vaccination against *Haemophilus infleunzae* type b and *Streptococcus pneumoniae* to the routine vaccination schedule, makes it difficult for clinicians to choose empirical treatment. Routine usage of sensitive diagnostic methods, such as PCR, in daily clinical practice would result in the choice of appropriate antibiotic therapy. In our study, the PCR results were available at the end of the study period; hence, we could not evaluate the effects of the PCR results on treatment selection, change, or duration. On the other hand, the inability to provide antibiotic sensitivity and high costs are the most important limitations of PCR [18]. In this study, we detected *S. aureus* in eight children with SA with PCR; however, we have no information about the methicillin susceptibility. The lack of antibiotic sensitivity results indicates the necessity of culture techniques especially nowadays as antibiotic resistance among microorganisms is increasing. Also, our sample size is not enough to draw any conclusions about the seasonal distribution of pathogens.

## 5. Conclusions

The results of this study provide important information about the etiology of SA in children. *S. aureus* is the main pathogen; *K. kingae* can be the etiologic agent, especially in patients under the age of 4. The use of advanced diagnostic approaches, like real-time PCR, with standard culture- methods in daily clinical practice will improve diagnosis and provide significant benefits in disease management and treatment. However, a great percentage of culture and PCR-negative cases demonstrate that all patients with acute monoarthritis should be evaluated with all combined clinical and laboratory findings.

## Figures and Tables

**Table 1:** Demographic and clinical characteristics of children with SA.

	n = 75
**Gender (Boys/Girls)**	45/30
**Age (months; mean ± SD)**	86.3 ± 57.4
Season	
Autumn	30 (40%)
Winter	18 (24%)
Summer	17 (22.7%)
Spring	10 (13.3%)
**Underlying chronic disease**	14 (18.7%)
Asthma	5 (6.7%)
Cerebral palsy	2 (2.7%)
Congenital heart disease	1 (1.3%)
Crohn’s disease	1 (1.3%)
Type 1 diabetes mellitus	1 (1.3%)
Hemophilia A	1 (1.3%)
Juvenile rheumatoid arthritis	1 (1.3%)
Meningomyelocele	1 (1.3%)
Hydronephrosis	1 (1.3%)
**Complaints at admission**	
Joint pain	63 (84%)
Joint swelling	56 (74.6%)
Decreased range of motion	47 (62.6%)
Erythema	27 (36%)
**Involved joints**	
Knee	58 (77.4%)
Hip	11 (14.7%)
Ankle	4 (5.3%)
Elbow	1 (1.3%)
Knee/ankle	1 (1.3%)

**Table 2:** Joint fluid culture and rt-PCR results in children with SA.

	Total n = 33	*Staphylococcus aureus* n = 22		*Kingella kingae* n = 3	*Streptococcus pyogenes* n = 4	*Salmonella* spp. n = 1	*Streptococcus pneumoniae* n = 1	Others n = 2
SF culture	7	MSSA 2	MRSA 1	0	1	1	0	2
SF PCR	14	8 *		3	3	0	0	0
SF culture + PCR	12	MSSA 6	MRSA 5	0	0	0	1	0

MSSA: methicillin-sensitive *Staphylococcus aureus*, MRSA: methicillin-resistant *Staphylococcus aureus*, PCR: polymerase chain reaction, SF: synovial fluid. * PCR analysis of synovial fluid did not perform methicillin susceptibility.

**Table 3:** Pathogens distributions according to age group, season, and joint involvement.

	*Staphylococcus aureus* n = 22	*Streptococcus pyogenes* n = 4	*Kingella kingae* n = 3	*Salmonella* spp. n = 1	*Streptococcus pneumoniae* n = 1	Others n = 2
**Age**						
3 months–5 years	7	3	3	1	1	-
>5 years	15	1	-	-	-	2
**Season**						
Autumn	4	2	2	-	1	-
Winter	7	2	-	-	-	-
Summer	7	-	1	-	-	2
Spring	4	-	-	1	-	-
**Involved joints**						
Knee	16	4	3	1	1	2
Hip	5	-	-	-	-	-
Ankle	1	-	-	-	-	-

## Data Availability

Data is unavailable due to privacy or ethical restrictions.
